# Hyperbaric oxygen therapy in managing systemic inflammatory response syndrome caused by ischemia-reperfusion injury following hand replantation and long-term outcomes: A report of two cases

**DOI:** 10.1016/j.amsu.2020.10.023

**Published:** 2020-10-21

**Authors:** Mendy Hatibie Oley, Maximillian Christian Oley, Andi Asadul Islam, Mochammad Hatta, Muhammad Faruk, Albertus Djarot Noersasongko, Harry Soenaryo, Deanette Michelle R. Aling, Jane Angela Kalangi, Marcella Tirza Tulong

**Affiliations:** a Plastic Reconstructive and Aesthetic Surgery Division, Department of Surgery, Faculty of Medicine, University Sam Ratulangi, Manado, Indonesia; b Plastic Reconstructive and Aesthetic Surgery Division, Department of Surgery, R. D. Kandou Hospital, Manado, Indonesia; c Hyperbaric Centre Siloam Hospital, Manado, Indonesia; d Division of Neurosurgery, Department of Surgery, Faculty of Medicine, University Sam Ratulangi, Manado, Indonesia; e Division of Neurosurgery, Department of Surgery, R. D. Kandou Hospital, Manado, Indonesia; f Department of Neurosurgery, Faculty of Medicine, Hasanuddin University, Makassar, Indonesia; g Clinical Microbiologist Program, Faculty of Medicine, Hasanuddin University, Makassar, Indonesia; h Department of Surgery, Faculty of Medicine, Hasanuddin University, Makassar, Indonesia; i Division of Orthopedic and Traumatology Surgery, Department of Surgery, Faculty of Medicine, University Sam Ratulangi, Manado, Indonesia

**Keywords:** Hand replantation, HBOT, Ischemic reperfusion injury, SIRS, Case report

## Abstract

**Introduction:**

Ischemia-Reperfusion Injury (IRI) is a complication following the reperfusion of ischemic tissues; it requires immediate treatment, as it can lead to severe infection and tissue death. The purpose of this study was to demonstrate the ability of Hyperbaric Oxygen Therapy (HBOT) to treat SIRS (Systemic Inflammatory Response Syndrome) caused by IRI and to provide long-term functional assessment for a period of up to 5 years.

**Case presentation:**

Two cases of avulsions of the hand at the levels of the wrist joint and the medial third forearm, severed by machetes. Both patients were male and in their twenties. Hand replantation was carried out after 30 minutes (medial third forearm case) and 11 hours (wrist joint case) of ischemic time. A couple of days after surgery, both patients experienced SIRS as a result of IRI. The patients were brought to the hyperbaric chamber and received 3 consecutive 90-min sessions of HBOT at 2.4 ATA 3 days in a row. The outcomes were compared in a table with each patient's vital signs and laboratory results, both before and after HBOT. A significant improvement was seen at the follow-ups in vital signs and laboratory results for both patients after HBOT administration. Long-term follow-up also showed satisfying results for hand function, proven by low DASH (Disabilities of the Arm, Shoulder, and Hand) scores.

**Conclusion:**

HBOT was able to treat SIRS in both patients. Favorable long-term hand function results signify successful extremity replantation.

## Highlights

• Ischemia-Reperfusion Injury (IRI) is a complication following the reperfusion of ischemic tissues.
• Two cases of avulsions of the hand that had underwent hand replantation had experienced systemic inflammatory response syndrome (SIRS) due to IRI.
• Administration of HBOT has been proven to be an effective treatment for post-hand replantation SIRS.

## 1 Introduction

Ischemia Reperfusion Injury (IRI) is a critical condition in which tissue damage is further worsened by the restoration of blood flow; it is often seen in patients with traumatic amputations after blood flow is restored to the affected area [1]. Traumatic amputations occasionally require replantation, or reattachment of a completely amputated body part, which restores both arterial inflow and venous outflow to the tissue [2]. When oxygen is restored, it may cause a number of effects: a burst of oxidative injury, facilitated by the production of reactive oxygen species (ROS) and the reduction of antioxidant reserves in cells; release of inflammatory mediators; arterial vasoconstriction; thrombosis; and leucocyte-endothelial cell adhesion [1]. This can cause Systemic Inflammatory Response Syndrome (SIRS), which eventually leads to multiple organ failure and death [3]. Therefore, reestablishing adequate cell perfusion is essential in order to prevent further cell damage. Hyperbaric Oxygen Therapy (HBOT) utilizes high-pressure oxygen to create a therapeutic effect for IRI in cells. It diffuses oxygen intracellularly, which promotes neovascularization and recovers post-ischemic tissues [4]. In this study, the DASH (Disabilities of the Arm, Shoulder and Hand) was used to monitor long-term hand function, because this tool is able to accurately assess impact and a person's ability to function due to an impairment [5]. This Case has been reported in line with the SCARE 2018 guidelines [6]**.**

## 2 Case presentation

### 2.1 Patient 1

**Fig. 1:**
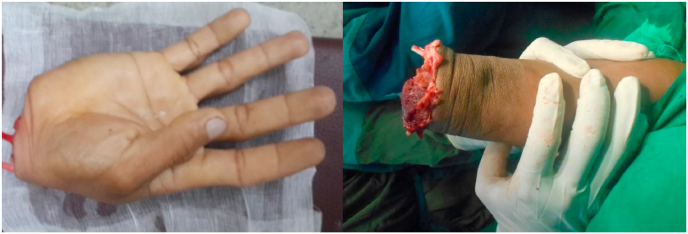
The right hand severed at the wrist joint level.

**Fig. 2:**
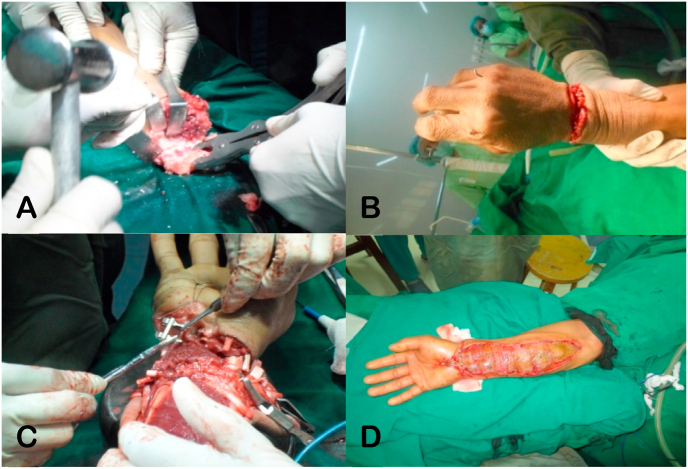
Replantation surgery of the right hand: A) Shortening osteotomy of approximately 1cm of the radius, ulna, and carpal bones; B) K-wire fixation; C) Tendon repair, arterial venous anastomosis, and nerve repair; D) Full-thickness skin graft.

A 29-year-old male presented with a clean cut at the right wrist by a machete. The incident happened in a rural village, and the patient was brought to a local clinic with his severed hand wrapped in banana leaves before he was referred to a district hospital for resuscitation and early wound management. He was then referred to Kandou Hospital Manado; his arm was wrapped in gauze, while the severed hand was placed in a plastic bag without ice—meaning that the amputated extremity had a total of 11 hours of warm ischemic time. Upon physical examination, we found traumatic amputation at the right wrist joint, and the severed extremity had an unpleasant smell (Fig. 1). Laboratory test values were within normal limits. We (the reconstructive & aesthetic plastic surgeon, neurosurgeon and orthopedic surgeon) performed surgical treatments on this patient included shortening osteotomy of the radius, ulna and carpal bones, k-wire fixation, fasciotomy, tendon repair, arteriovenous anastomosis, nerve repair, and full-thickness skin graft (Fig. 2). In the ICU, the patient's arm was raised in order to reduce swelling.

**Fig. 3:**
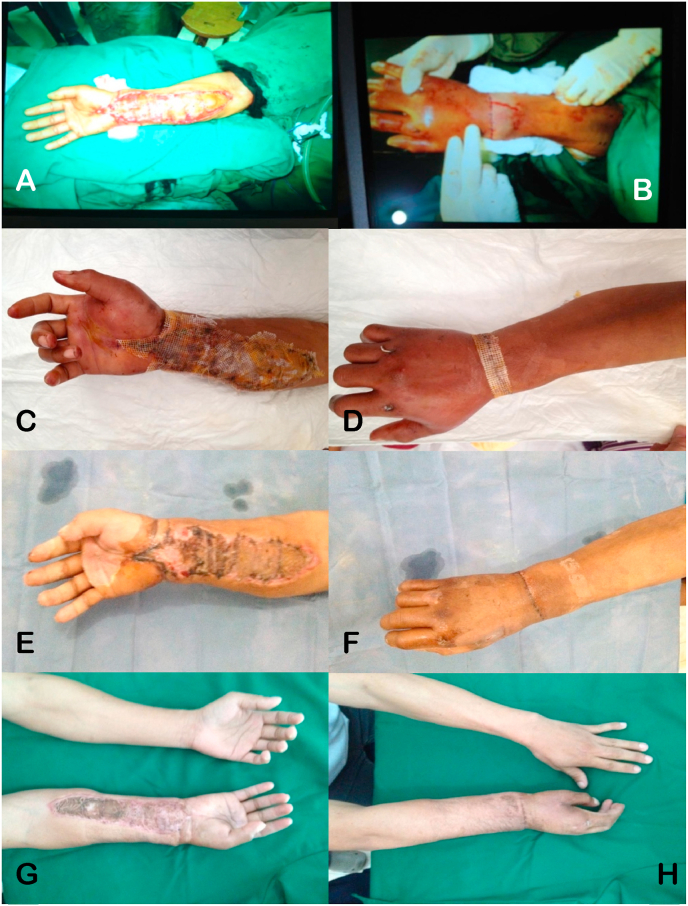
The changes in the replanted right hand, over a period of 2 months: A) and B) Post-replantation of the right hand; C) and D) The right hand after 3 HBOT sessions; E) and F) The right hand at one-month post-replantation; G) and H) The right hand at two months post-replantation.

**Fig. 4:**
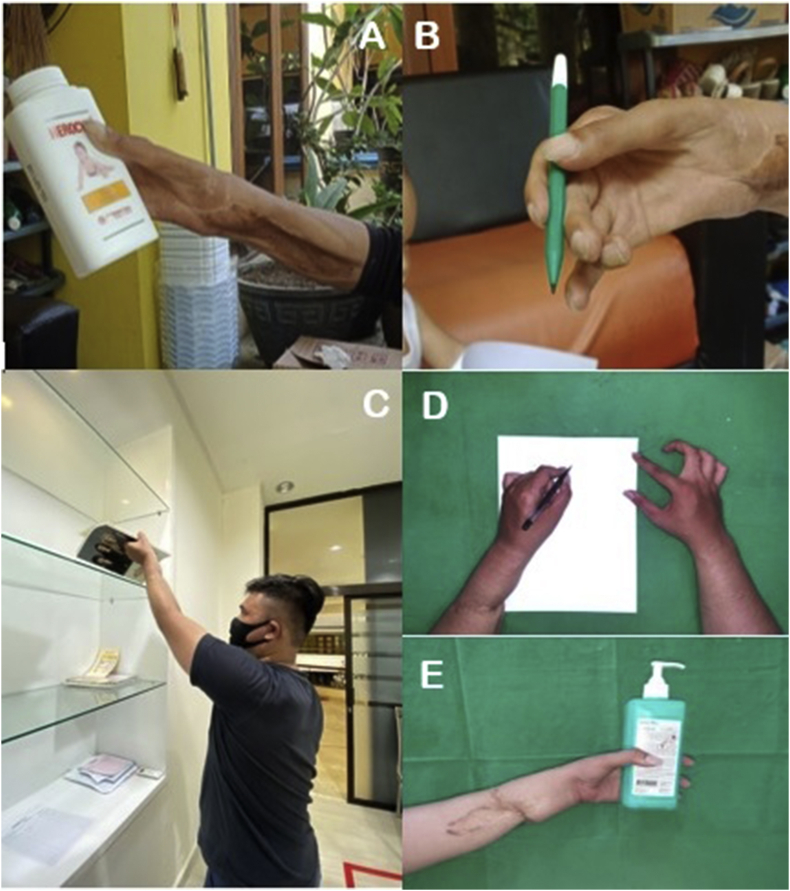
Patient 1 and Patient 2 ability to use the upper extremity, 5 years post-replantation: A) Right hand grip, B) Patient holding a pen with his right hand; C) Patient placing an object on a shelf above the head; D) Patient writing with the left hand; E) Left-hand grip.

The following day, the patient experienced severe pain in his right arm (Visual Analog Scale 8–9), a Glasgow Coma Scale (GCS) score of 12, fever (40 °C), hypertension (150/100 mmHg), and an increased heart rate (105 bpm). His leucocyte count was 26.0 x 10^9^/L, and both liver and kidney functions had increased (see Table 1). HBOT was administered immediately after these measurements were obtained. The patient was given 3 sessions of HBOT 3 days in a row, at a duration of 90 minutes per session, at 2.4 ATA. Assessment after 3 sessions of HBOT showed significant improvements on both clinical and laboratory tests. A 2-month post-surgery follow-up showed a mild scar at the volar surface of the lower arm (Fig. 3). After 6 months, the patient was able to utilize his right hand, although his right wrist was still unable to perform flexion and extension. Currently, at 5 years after surgery, the patient is able to comfortably ride his motorcycle, gripping an object, writing and is satisfied with his progress (Fig. 4a, and 4b).

**Table 1:** Patient follow-up before and after HBOT administration.

Patient		BP (mmHg)	HR (bpm)	RR (bpm)	Temperature (°C)	Leucocyte count (x 10^9^/L)	GCS	Urea and creatinine levels (mg/dl)	AST and ALT (IU/l)
1	Before HBOT	150/100	105	26	40 °C	26.0	13	100, 1.9	50, 55
	After HBOT	110/80	84	20	37 °C	11.0	15	60, 1.0	30, 20
2	Before HBOT	130/90	110	28	39 °C	20.0	13	75, 1.5	40, 40
	After HBOT	120/80	80	22	36.8 °C	9.0	15	50, 0.9	35, 30

**Abbreviation:** BP = blood pressure; HR = heart rate; RR = respiration rate; GCS = Glasgow Coma Scale; AST = aspartate transaminase; ALT = alanine aminotransferase; bpm = beats per minute and/or breaths per minute.

### 2.2 Patient 2

**Fig. 5:**
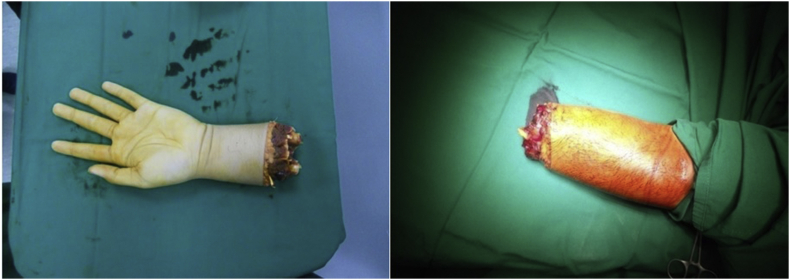
The severed left hand, at the medial third forearm level.

**Fig. 6:**
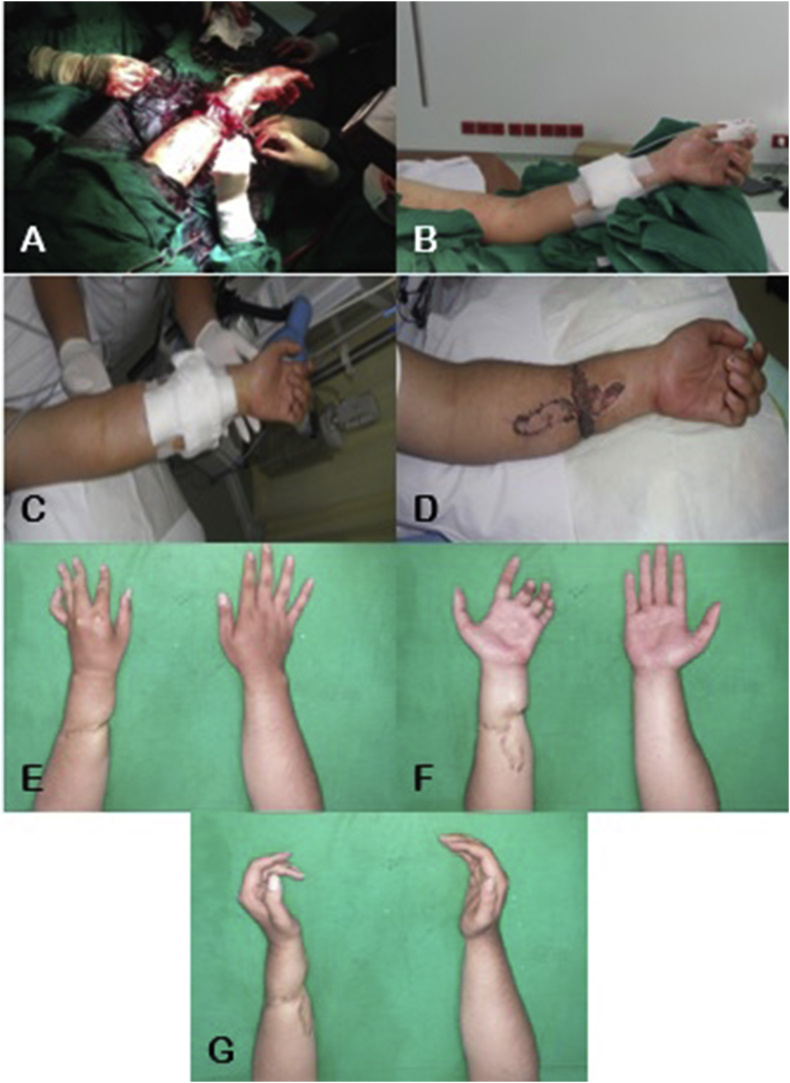
Intraoperative and post-operative pictures of the left forearm: A) Replantation surgery of the left forearm; B) The left forearm post-replantation; C) The left forearm after 3 HBOT sessions; D) The left forearm at 2 weeks post-replantation; E), F) and G) The left forearm at 5 years post-replantation.

A 20-year-old male presented with a severed left hand at the medial third forearm level, caused by a machete. The patient traveled without delay to Siloam Hospital Manado, arriving 30 minutes after the incident. The severed hand was brought along by the patient, carried without a container. Physical examination showed a traumatic amputation at his mid-forearm (Fig. 5). Laboratory test results were within normal range. He was brought in for surgery and received shortening osteotomy of the radius, ulna, and carpal bones, fixation with titanium plate and screw, fasciotomy, tendon repair, and arteriovenous anastomosis (Fig. 6a). Two days post-surgery, the patient felt numbness in his left arm (Fig. 6b) and displayed an increased respiratory rate (28 times per minute), a GCS score of 13, increased heart rate (110 bpm), and fever (39 °C), but had controlled blood pressure. Laboratory test results showed a slight increase in his leucocyte count (20.0 x 10^9^/L) and slightly elevated liver and kidney functions (see Table 1). He was taken to the hyperbaric chamber and was given 3 sessions of HBOT 3 days in a row (Fig. 6c), at a duration of 90 minutes per session, at 2.4 ATA. His condition vastly improved after the first HBOT session. Two weeks after replantation (Fig. 6d), the surgical wound appeared to have healed. After 5 years, although the replanted extremity was cold-intolerant and the patient was unable to extend his third, fourth, and fifth digits (Fig. 6e, f, and 6g), he was still able to use his hand to perform daily activities such as placing an object , writing, and gripping an object (Fig. 4c, d and 4e).

After experiencing SIRS following hand replantation, both patients’ symptoms receded once HBOT was administered. Table 1 displays a follow-up of both patients before and after HBOT.

Prior to HBOT administration, both patients began to experience symptoms of SIRS, including fever, increased heart rate, increased respiration rate, altered mental status, increase in leucocyte count, and increase in liver and kidney functions. After HBOT, symptoms in both patients receded, and laboratory test results were restored to normal values.

Over a period of 5 years post-replantation, hand function was assessed using the DASH scoring system (Table 2). Patient 1 and Patient 2 had final DASH scores of 25 and 23.5, respectively.

**Table 2:** DASH Score Assessment over 5-year period.

DASH Score				
Patient	1 month	2 months	1 year	5 years
1	50	40	35	25
2	55	43.5	30	23.5

## 3 Discussion

Patients with traumatic amputations experience life-threatening complications even after the main injuries have been successfully treated. In the Case of traumatic extremity amputations, despite successful replantation of the amputated body part, it is essential to monitor the patient's condition post-replantation, as the restoration of blood flow to ischemic tissues can lead to reperfusion injury [2]. Reperfusion injury occurs when blood flow to ischemic tissues is reestablished and causes a litany of events leading to oxidative stress in cells, systemic inflammatory response, multiple organ failure, and eventually death [3].

Hand replantation after traumatic amputations reconnects the severed hand in order to restore its arterial and venous flow. In general, patients with amputations involving upper limbs are candidates for replantation. It is essential to obtain a history of the patient, including the patient's age, hand dexterity, occupation, pre-existing systemic diseases, and a description of the mechanism of the injury. Moreover, chest x-rays, electrocardiogram, complete blood count, electrolyte count, blood type, cross-matching, and radiographs of the amputated section and proximal limb should also be performed [7]. Treatment of the amputated extremity is also vital to preserving its important structures. The amputated extremity should be cooled down immediately, since the forearm can tolerate up to 10 hours of cold ischemia and only 4–6 hours of warm ischemia [8]. The duration of ischemia and the temperature in which the amputated extremity is preserved can determine the severity of IRI; therefore, it is imperative to keep the amputated segment is cool temperatures [9]. In our study, Patient 1 had a warm ischemic time of 11 hours, which makes replantation theoretically unfeasible. However, since the blood vessels were not clamped, they were preserved, making it easier for us to reattach the hand. Nayak et al. reported cases of hand replantation involving 6–8 hours of warm ischemia and concluded that arterialization prior to replantation should be performed immediately, in order to delay tissue necrosis and enable limbs to survive after long warm ischemic time [10]. The replantation technique includes irrigation and debridement, identification, dissection, marking of nerves and blood vessels, bone shortening, internal fixation, extensor tendon grafting, flexor tendon grafting, arterial, vein and nerve grafting, and skin graft (to close the wound) [1,11].

**Fig. 7:**
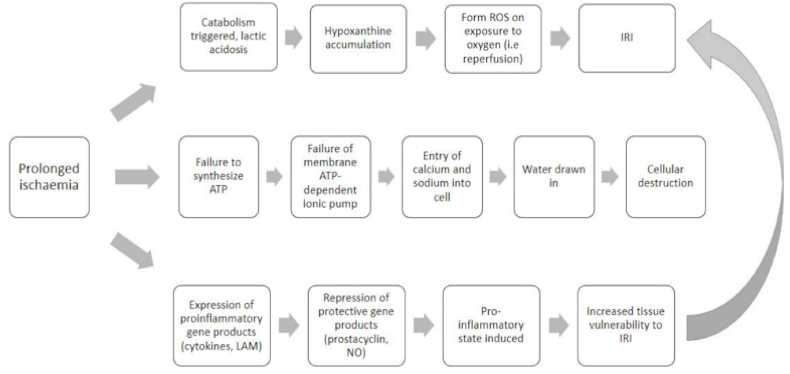
Cellular effects of prolonged ischemia [13].; Abbreviations: ATP = Adenosine Triphosphate, ROS = Reactive Oxygen Species, IRI = Ischemia Reperfusion Injury, LAM = Lipoarabinomannan, NO = Nitric oxide.

After replantation and a return of blood flow to the reattached extremity, more oxygen will enter the tissue with the goal of promoting cell recovery. However, prolonged ischemia will cause cellular alterations that are detrimental to the tissue (Fig. 7) [2]. In a physiological state, ROS plays a role in the body's defense mechanisms against pathogen as well as in tissue repair. ROS can have both beneficial and harmful effects on cells. The beneficial effects of ROS include initial wound protection, leucocyte recruitment, tissue repair, stimulation of revascularization, and mediation in the wound healing process [4]. However, ROS levels are controlled by intracellular antioxidants; when blood flow is returned to hypoxic tissues, oxygen molecules will bind to each other, which—given the absence of a supply of local antioxidants—will cause a surge in ROS. In the end, instead of promoting cell recovery, the excessive amount of ROS can cause cellular damage [11]. Moreover, prolonged ischemia triggers inflammation and influences the wound healing process. Inflammation occurs when phagocytes (such as leucocytes and neutrophils) travel to the wound site to debride the wound [4]. When these inflammatory cells consume the pathogen, they produce phagolysosome, which in turn activates a phagocytic enzyme called NADPH; at that point, NADPH undergoes oxidation and becomes superoxide radicals. After the dismutation of hydrogen peroxide (H_2_O_2_), toxic molecules such as hydroxyl radical and hypochlorous acid (forms of ROS) are formed [2]. Subsequently, oxygen reacts with pre-existing free radicals, causing IRI [12].

Furthermore, the production of macrophage-stimulating factors by CD4^+^ T lymphocytes activates local macrophage cells and the release of cytokines, which together with ROS induce oxidative stress and increase endothelial adhesion molecules in organs. In addition, low nitric oxide levels result in vasoconstriction, which—along with the increased expression of adhesion molecules—entrap platelets and neutrophils in local vascular structures. Microcirculatory insufficiency leads to ischemia and necrosis, macrophage cell activation, and the cyclic release of ROS and inflammatory cytokines. All these occurrences lead to systemic inflammatory response of the body [7]. One study found a significant increase in plasma concentrations of urea, creatinine, aspartate transaminase, alanine transaminase, and lactic dehydrogenase in Wistar rats that underwent reperfusion compared to rats in the control group (*p* < 0.001) [3]. Ischemia can also result in cellular destruction caused by the failure of ATP-dependent sodium potassium pump, hindering ionic exchange. When ionic exchange is hindered, water is drawn intracellularly, causing cells to swell and membranes to weaken. Consequently, cells become susceptible to destruction [13].

SIRS is assessed by the Sequential Organ Failure Assessment (SOFA) or quick SOFA (qSOFA). A SOFA score of <9 marks a low mortality risk, 9–11 indicates a moderate risk, while >11 shows a high risk. Parameters in the SOFA include respiratory rate, platelet count, liver and bilirubin functions, cardiovascular function, mental alertness, and kidney function, with each parameter given a score of 1–4. In contrast, qSOFA uses only 3 parameters: respiratory rate of ≥22/min, altered mental status, and a systolic blood pressure of ≤100 mmHg; each parameter is given a score of 1. A qSOFA score of ≥2 indicates a high risk of mortality [14]. Our patients had qSOFA scores of 2 which means that IRI may have induced SIRS in our patients. As seen here, the use of HBOT was proven to improve SIRS symptoms in our patients.

In the field of plastic surgery, HBOT has been commonly used to accelerate wound healing. It is particularly recommended for Gustilo 3B and 3C injuries, although less severe wounds may also be treated with HBOT [15]. Generally, HBOT has three mechanisms of actions: increasing oxygen partial pressure, stimulating favorable vascular effects, and augmenting physical pressure. Due to the increase in arterial and capillary oxygen tension, oxygen is delivered through endothelial diffusion in order to increase oxygen tension in tissue. The increased oxygen levels create favorable effects that support wound healing and reduce infections—effects that include neovascularization, increase of ROS in leucocytes, increase of dissolved oxygen, tissue oxygenation, and inhibition of endothelial adhesion by leucocytes [4]. HBOT is useful when limb replantation demonstrates marginal circulation. According to Nylander et al., HBOT significantly reduces phosphorylase activity, a sensitive marker for muscle breakdown, in the post-ischemic phase [16]. Hyperbaric oxygen therapy is administered at 2.5 ATM for 45 minutes, and three treatments are often required. For adequate oxygen delivery, partial oxygen pressure and oxygen tension must be higher than the atmospheric pressure of 760 mmHg under 1 ATA; in a hyperbaric chamber, the pressure is expanded to at least 1433 mmHg under 2 ATA, allowing oxygen to diffuse in the bloodstream [4].

Significant improvements with HBOT in skin grafts and flaps have been reported since 1967. The Undersea & Hyperbaric Medical Society (UHMS) recommends twice-daily treatments at 2.0–2.5 ATA for 90–120 minutes and decreasing this regime to once-daily when the graft or flap has stabilized. One study showed that HBOT combined with hydrogen-rich saline is an effective way to improve flaps or reperfusion injury in rats [17]. In addition, a literature review presented several cases wherein HBOT was proven to be beneficial in treating crush injuries wounds and flap survival [15]. In our cases, both patients experienced post-replantation SIRS due to reperfusion injury, and both showed improvement of symptoms and laboratory test results with HBOT administration.

Post-replantation surgery follow-up of hand function is needed to assess a patient's levels of disability and satisfaction. The DASH is a tool used to measure upper extremity disability using a 30-question questionnaire. Several studies have proven this scoring system to be accurate in evaluating long-term functional outcomes after extremity and digital replantation [18,19]. The score ranges from 0 to 100, with 0 indicating no disability and 100 indicating greater disability [18]. Both our patients had low final DASH scores of 25 and 23.5, meaning that their disability did not limit their ability to perform daily tasks and that they were satisfied with the way their replanted extremity functioned.

In the weeks and months immediately following replantation, additional surgery may be required to improve the function of the replanted limb [16]. Unfortunately, due to distance from the hospital and transportation issues, after the first year, our patients were lost to follow-up appointments until 5 years later. As a result, we were not able to perform further reconstructive surgeries on our patients. Nevertheless, both patients were content with their progress after 5 years.

## 4 Conclusion

Administration of HBOT has proven to be an effective treatment for post-hand replantation SIRS, which in both of our cases was caused by reperfusion injury. Hand function in both patients showed favorable outcomes at a 5-year follow-up.

## Provenance and peer review

Not commissioned, externally peer reviewed.

## Declaration of competing interest

The authors declare that they have no conflicts of interests.
